# Satisfaction and tolerability using virtual reality (VR) as adjunctive treatment during flexible bronchoscopy: a randomized control trial

**DOI:** 10.1186/s12890-023-02304-y

**Published:** 2023-01-10

**Authors:** Ian Victor Sooriyaghandan, Mas Fazlin Mohamad Jailaini, Nik Nuratiqah Nik Abeed, Boon Hau Ng, Andrea Ban Yu-Lin, Shamsul Azhar Shah, Mohamed Faisal Abdul Hamid

**Affiliations:** 1 Respiratory Unit, Faculty of Medicine, Universiti Kebangsaan Malaysia, Jalan Yaacob Latif, Bandar Tun Razak, 56000 Kuala Lumpur, Malaysia; 2 Department of Community Health, Faculty of Medicine, Universiti Kebangsaan Malaysia, Kuala Lumpur, Malaysia

**Keywords:** Flexible bronchoscopy, Satisfaction, Tolerability, Virtual reality (VR) device

## Abstract

**Background:**

Patient comfort during invasive and therapeutic procedures is important. The use of virtual reality (VR) devices during flexible bronchoscopy (FB) as a method of distraction to increase patient tolerability and improve satisfaction has not been investigated. We aim to assess the satisfaction and tolerability of participants undergoing FB with or without VR.

**Methods:**

This was a single-center, open-label study on patients undergoing bronchoscopy, randomized into the control and interventional (VR) groups. The control group received standard care during FB. The interventional group was given a VR device during FB showing nature videos with soothing instrumental music. Pain, breathlessness, and cough were evaluated using a 10 cm visual analogue scale administered before and after FB. Anxiety was assessed using the State-Trait Anxiety Inventory. Satisfaction questionnaire (5-point Likert scale) was given to participants post FB.

**Results:**

Eighty participants enrolled, 40 in each arm. Median (IQR) satisfaction score in the VR group was 5.0 (3.0–5.0), and in the control group was 4.0 (3.0–5.0); (*p* < 0.001). Breathlessness, cough, and anxiety post FB were significantly less severe in the interventional group (*p* = 0.042, *p* = 0.001, *p* < 0.001), but the pain was not significantly different (*p* = 0.290).

**Conclusion:**

VR used during FB led to better participants' satisfaction and tolerability (breathlessness and cough). There was a significantly lower anxiety score in the VR group.

## Introduction

Flexible bronchoscopy (FB) is a necessary procedure used worldwide to diagnose and treat the disease of the lungs and airways [1]. The procedure may be performed in an endoscopy suite, the operating room, the emergency department, or at the bedside in the intensive care unit [2]. FB is a safe procedure widely used to manage patients with respiratory diseases [3]. FB has nearly zero mortality, and major complications are rare [4]. Besides being safe and accurate, the patient's comfort during the procedure is the primary concern of the medical and endoscopy staff [5].

FB can be a frightening and painful experience for the patient [6]. There is room to improve pain management during FB, as many patients experience pain despite the common use of premedication analgesics and sedatives [7, 8]. In addition, because these medications have side effects, including respiratory depression and cardiovascular instability, it would be useful to develop non-pharmacologic approaches in improving the patient experience with painful procedures [9]. Using less medication may speed recovery post-procedure and facilitate the timely discharge of patients from the hospital.

Non-pharmacologic practices, such as guided imagery, hypnosis, and distraction, have improved patients' experiences during stressful or painful medical procedures [10, 11]. Distraction therapy is a technique in which sensory stimuli are provided to patients to divert their attention from an unpleasant experience [12]. The use of nature scenes and sounds is an effective tool for distraction and has been successful in various patient settings, including perioperative care, phlebotomy, and burn care [10, 13, 14]. However, the benefit of non-pharmacologic approaches to analgesia during FB has not been evaluated adequately. A VR device is an apparatus with a head-up display (HUD) that projects a video and sound. The device is aimed to replace the patient’s natural environment with virtual reality content.

In Malaysia, FB has been well established as a means of diagnosis and treatment for many respiratory diseases; however, there are no studies on non-pharmacological methods to improve patients' experience during bronchoscopy. The use of conventional analgesia and sedation is considered the standard of care during FB. In Universiti Kebangsaan Malaysia Medical Centre (UKMMC), fentanyl and midazolam are used routinely as sedation during FB. There was a previous study using a combination of hypnosis and VR technology during flexible bronchoscopy [15]. However our study is unique as this is the first randomized control trial comparing VR to standard practice, as a method of distraction to reduce anxiety and improve satisfaction in a patient undergoing bronchoscopy. This study explores the use of VR in participants' satisfaction, tolerability and anxiety during FB.

## Methods

### Study design

This was an open-label, prospective interventional single-center study conducted on participants undergoing FB under the Respiratory Unit, UKMMC between May 2022 and August 2022. The study was approved by the Research Ethics Committee, University Kebangsaan Malaysia, FF-2021–506, and registered with clinical trial number on 22/04/2022 (NCT05340907). Written informed consents were obtained from all participants prior to enrollment in this study according to international guidelines.

The sample size calculation was based on a study by Navidian et al.; using pain as a comparison between the intervention and control group, which is normally distributed with a standard deviation (SD) of 1.64 [16] The total sample size calculated was 80 (40 subjects in each group), allowing a 10% drop-out rate. The power of the study was designed at a level of 80%.

All participants who were planned for FB were recruited. We included the following: age 18 years and above; participants who could understand and give consent; and participants who had a negative covid polymerase chain reaction (PCR) or antigen test as per standard practice before bronchoscopy.

Participants were excluded if they were below the age of 18; unable to understand or give consent; those ventilated; not comfortable wearing VR device; who were unable to communicate (illiterate, had hearing impairment, mute, blind or had memory impairment); with craniofacial deformity; on sedative medication other than sedation given for bronchoscopy; undergoing other invasive examination planned alongside bronchoscopy; had bronchoscopy done in the past 12 months.

The primary outcome was to compare the satisfaction of participants undergoing FB with or without VR using a 5-point Likert scale satisfaction questionnaire (1 – very dissatisfied, 2-dissatisfied, 3-neutral, 4-satisfied, 5-very satisfied). The secondary outcome was to compare tolerability (pain, breathlessness, and cough) of participants undergoing FB with or without VR, using a 10 cm Visual analogue scale (VAS), ranging from 0 (no bother) to 10 (worst intolerable level). We also compared the anxiety of participants undergoing FB with or without VR using the State-Trait-Anxiety-Inventory (STAI) score. These questionnaires were administered in English or Malay, depending on the participants' preference.

STAI questionnaire is the “gold standard” for measuring procedural anxiety [17–19]. The reliability and validity of the STAI are well reported (Cronbach’s alpha = 0.896). It comprises separate self-report scales for measuring state and trait anxiety. The S-Anxiety scale (STAI Form Y-1) consists of twenty statements that evaluate how respondents feel “right now, at this moment. Scores on the S-Anxiety scale increase in response to physical danger and psychological stress and decrease as a result of relaxation. The scale has also been used to assess the level of S-Anxiety induced by stressful procedures and real-life stressors such as surgery or dental treatment [17]. Each question is a weighted score of 1–4 (not at all, somewhat, moderately so, very much so). The range of possible scores for the STAI varies from a minimum score of 20 to a maximum score of 80 on STAI-S subscales. We used the STAI questionnaires in English and a validated translated questionnaire in Malay in our study.

### Procedure

**Fig. 1:**
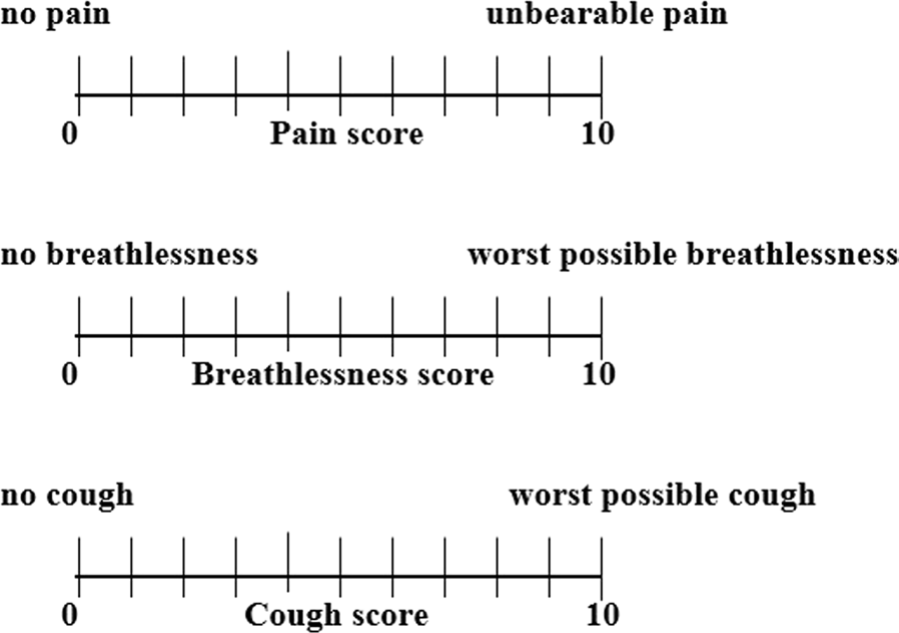
Visual analogue scales for pain, breathlessness, and cough anchored to 10 cm

Demographic data was collected prior to randomization, which included (age, gender, ethnicity, BMI, comorbidities) and indications for FB. The investigator then randomized with a block of 4 with random permutations of 2 groups: intervention (VR) and controlled group without VR. Before bronchoscopy, both groups were given a VAS questionnaire to assess their pain, breathlessness, and cough (Fig. 1). This was to determine the participant’s baseline score pre-procedure. The pain score was measured by the use of validated 10 cm VAS, a 10 cm line anchored with “no pain” at 0 cm and “unbearable pain” at 10 cm. The breathlessness VAS was measured with a similar scale which was anchored with “no breathlessness” at 0 cm, and “worst possible breathlessness” at 10 cm. The cough level VAS was anchored with “no cough” at 0 cm and “worst possible cough” at 10 cm.

After completing the questionnaires, both groups received topical anesthesia administered intranasal and intra-pharyngeal. The anesthesia comprised of intranasal lignocaine HCL 2% jelly (XYLOCAINE® 2% JELLY) followed by Lignocaine Hydrochloride Spray 10% (XYLOCAINE® SPRAY). The bronchoscopy was done per standard protocol for participants in the control group.

**Fig. 2:**
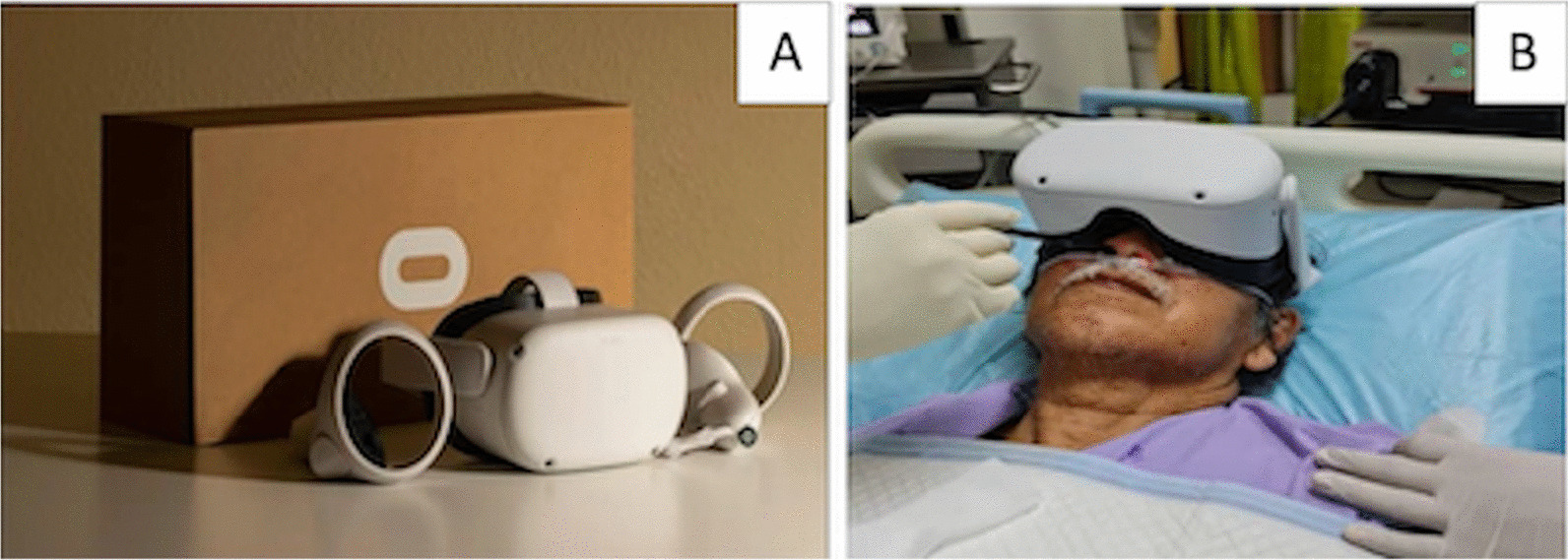
Oculus Quest 2 Virtual Reality Device (**A**). Participant undergoing bronchoscopy using VR device (**B**) (consent for publication of photo taken from a participant)

Participants in the interventional group were given the virtual reality (VR) device to wear, which was the Oculus Quest 2 (Oculus, China)—(Fig. 2), before bronchoscopy. They were shown videos of nature sceneries accompanied by soothing instrumental music. The level of immersion that participants received were 3-dimensional video natures scenes from various countries coupled with instrumental music via surround sound speakers. The participants received 10 min of screening time before undergoing FB, and the device was removed upon completion of bronchoscopy. The VR device was sanitized before and after each use, and participants were given disposable hygiene covers to prevent the risk of pathogen transmission. Post FB, participants were observed in the bronchoscopy suite for at least 4 h.

Both groups were given sedation as per standard protocol pre-FB. Participants were given sedation of midazolam 1–2 mg, (3 min before FB), with a supplemental titrating doses of 1-2 mg midazolam as needed, and titrating doses of fentanyl from 12.5mcg upwards. For those in the intervention group, the sedations were administered 7 min into screening time (3 min before FB), and at the end of 10 min, bronchoscopy was performed. During FB both groups also received topical anesthesia of the airway using Lignocaine HCL INJECTION 2% (20 mg/ml; not exceeding 8 mg/kg body weight), which was administered via the working channel port of the bronchoscope. Throughout the procedure in both groups; constant communication were maintained between participants and bronchoscopy staff.

The FB was performed by the same independent bronchoscopist with a 3-year experience in performing bronchoscopy; using the PENTAX Medical Video Bronchoscope EB-J10 (Pentax Medical, Japan). The FB was done via the anterior approach with the head of the bed propped up at 30^0^.

**Fig. 3:**
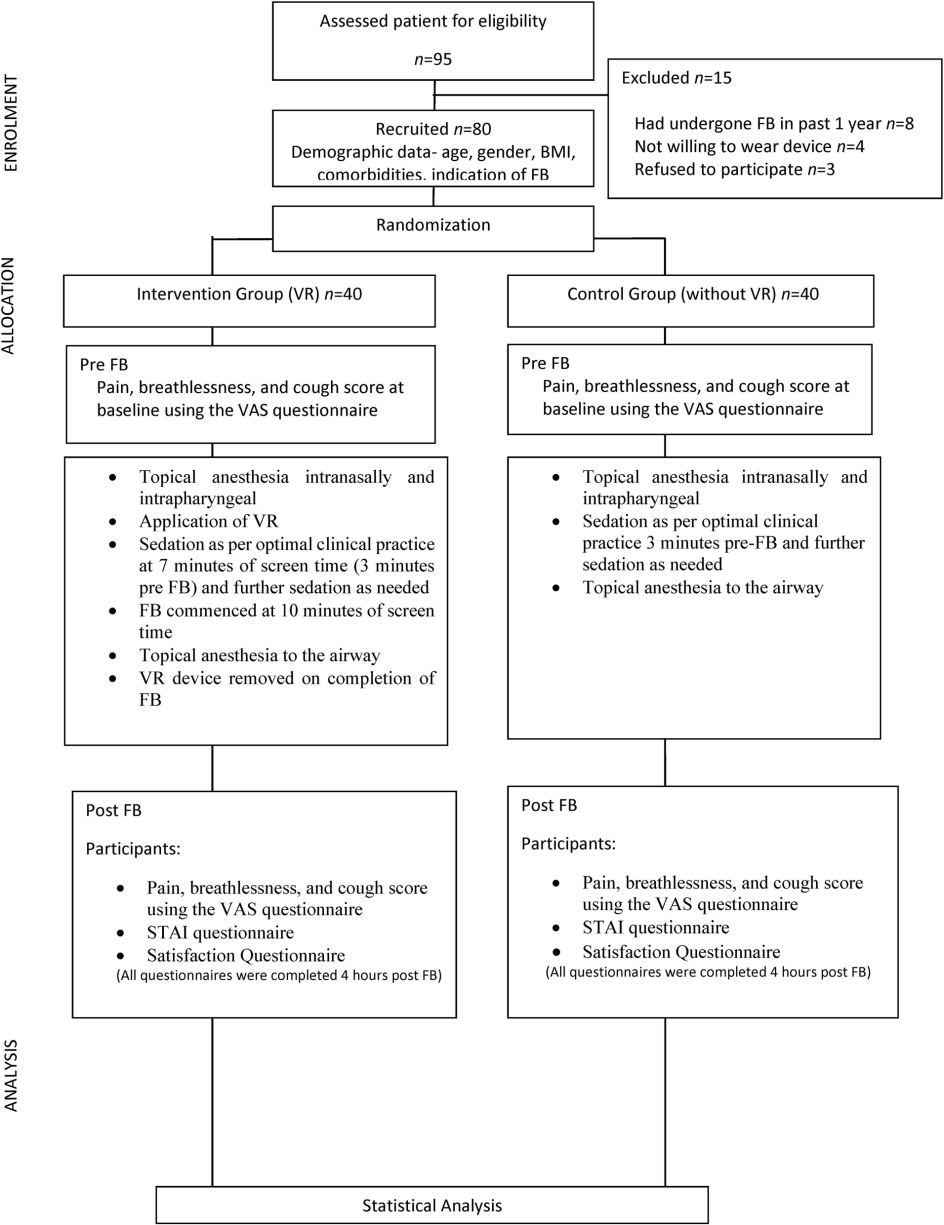
CONSORT flow diagram

Post-procedure, all participants were given VAS, STAI, and satisfaction questionnaires. These were completed by the participants at 4 h post FB. This was to ensure that there was no residual effect of sedation and that participants were fully conscious. The study design and CONSORT flow diagram, as shown in Fig. 3.

### Statistical analysis

All statistical analyses were performed using Statistical Package for Social Sciences version 27.0 (SPSS Inc, Chicago, IL, USA). Variables with normal distribution were expressed as mean ± standard deviation. Variables with non-normal distribution was expressed as median (interquartile) and range. Continuous variables with normal distribution were analyzed using student t-test while to compare between the two groups: interventional and non-interventional group. The categorical data was tested with Pearson Chi-square test and Fisher exact test. The results of the data between the two groups were analyzed using Wilcoxon Sign Rank Test or its equivalent non-parametric chi square test for parameter with non-normal distribution. Statistical significance declared when *p* < 0.05.

## Results

A total of 95 participants undergoing FB were screened between May 2022 and August 2022. Eighty participants fulfilled the inclusion criteria and consented to the study. Out of this, 40 participants were randomized into the intervention group and another 40 into the control group.

The baseline characteristics were similar between the interventional group and the control group. The median (IQR) age was 67(50.3–75.5) in the interventional group and 64 (41.8–69.8) in the control arm. A majority (50%) of the participant were aged 65 and above. When divided into different age groups, young adult, middle age, and older age groups, the age difference were not statistically significant (Table 1). The majority of participants were males (58.8%); this was true for both the interventional and control groups. More than half were Malays (55%), followed by Chinese (36.3%) and Indians (7.5%). The median BMI (IQR) was 23.3 (14.5–39) kg/m2 in the interventional arm and 23.8 (17.1–45.8) kg/m2 in the control arm with majority of patients belonging to normal BMI group (57.5%). Around 78.8% of the subjects had 1 or more comorbidities. Hypertension was the most common comorbidity (45.2%), followed by diabetes mellitus (35.2%) (Table 1).

**Table 1:** Demographic and baseline characteristics between interventional and control group (*n* = 80)

Variables	n (%)	Group		*p* value
		Intervention (n = 40)	Control (n = 40)	
Age, median (IQR)	80 (100)	67 (50.3–75.5)	64 (41.8–69.8)	0.223^c^
Age groups, n (%)				
Young adult (17–39)	12 (15.0)	3 (25.0)	9 (75.0)	0.159^a^
Middle age (40–64)	28 (35.0)	16 (57.1)	12 (42.9)	
Older age (> 65)	40 (50.0)	21 (52.5)	19 (47.5)	
Gender, n (%)				
Male	47 (58.8)	25 (53.2)	22 (46.8)	0.496^a^
Female	33 (41.3)	15 (45.5)	18 (54.5)	
Ethnicity, n (%)				
Malay	44 (55.0)	21 (47.7)	23 (52.3)	0.613^b^
Chinese	29 (36.3)	16 (55.2)	13 (44.8)	
Indian	6 (7.5)	2 (33.3)	4 (66.7)	
Others	1 (1.3)	1 (100.0)	0 (0.0)	
Comorbidities, n (%)				
Yes	66 (82.5)	30 (45.5)	36 (54.5)	0.056^a^
HT^©^	4 (5.0)	3 (75.0)	1 (25.0)	
DM®	3 (3.8)	2 (66.7)	1 (33.3)	
IHD^£^	1 (1.3)	1 (100.0)	0 (0.0)	
Dyslipidemia	2 (2.5)	2 (100.0)	0 (0.0)	
HT/DM/dyslipidemia	11 (13.8)	2 (18.2)	9 (81.8)	
HT/DM/IHD	1 (1.3)	0 (0.0)	1 (100.0)	
HT/IHD	1 (1.3)	0 (0.0)	1 (100.0)	
HT/DM	8 (10.0)	3 (37.5)	5 (62.5)	
HT/Dyslipidemia	6 (7.5)	3 (50.0)	3 (50.0)	
HT/DM/IHD/Dyslipidemia	5 (6.3)	1 (20.0)	4 (80.0)	
Others	24 (30.0)	13 (54.2)	11 (45.8)	
None	14(17.5)	10 (71.4)	4 (28.6)	
BMI (kg/m^2^), median (IQR)	80(100)	23.3(14.5–39.0)	23.8 (17.1–45.8)	0.324^c^
BMI groups (kg/m^2^)^@^ n (%)				
Underweight (< 18.5)	12 (15.0)	8 (66.7)	4 (33.3)	0.416^b^
Normal (18.5–24.5)	46 (57.5)	24 (52.2)	22 (47.8)	
Overweight (25.0–30.0)	13 (16.3)	5 (38.5)	8 (61.5)	
Obese (> 30.0)	9 (11.3)	3 (33.3)	6 (66.7)	

Statistical analysis was run using

^a^Chi-Square test of association

^b^Fisher-Exact test

^c^Mann Whitney-U test

^@^BMI based on WHO classification

©Hypertension

® Diabetes Mellitus

£ Ischemic Heart Disease

Using a 5-point Likert scale, the median (IQR) for satisfaction score in the interventional group was 5.0 (3.0–5.0), while in the control group the median(IQR) satisfaction score was 4.0 (3.0–5.0), (*p* < 0.001) (Table 2).

**Table 2:** Comparison of questionnaire score between interventional and control groups given pre and post bronchoscopy

Variables	Interventional (n = 40)	Control (n = 40)	*p* value
Pre-bronchoscopy comparison within interventional and control group (*n* = 80)			
VAS pain score (cm), median (IQR)	0.0 (0.0–0.0)	0.0 (0.0–0.0)	0.432^@^
VAS breathlessness score (cm)	0.0 (0.0–0.8)	0.0 (0.0–0.0)	0.473^@^
VAS cough score (cm)	1.0 (0.0–2.0)	1.0 (0.0–3.0)	0.907^@^
Post bronchoscopy comparison within interventional and control group (n = 80)			
VAS pain score (cm), median (IQR)	1.0 (0.0–2.0)	2.0 (0.0–2.0)	0.295^@^
VAS breathlessness score (cm)	0.5 (0.0–2.0)	2.0 (1.0–3.0)	**0.042**^@^
VAS cough score (cm)	2.0 (1.00–3.0)	4.0 (2.3–5.0)	**0.001**^@^
Satisfaction score (5-point Likert scale)	5.0 (3.0–5.0)	4.0 (3.0–5.0)	< **0.001**^@^
STAI score	29.5 (26.3–40.8)	36.5 (33.5–43.5)	< **0.001**^@^

^@^Mann–Whitney test; *p* value < 0.05 is significant (in bold)

Using a 10 cm VAS questionnaire, comparing the pain score pre-bronchoscopy between interventional and control groups, the median score was 0 in both groups (*p* = 0.432), post bronchoscopy analysis showed the median (IQR) score in the interventional group was 1.0 (0.0–2.0), the control group however had a median (IQR) of 2.0 (0.0–2.0), (*p* = 0.295) (Table 2).

The breathlessness score pre bronchoscopy comparing the interventional and control group was analyzed; the median for both groups were 0 (*p* = 0.473), post bronchoscopy, the interventional group had a median (IQR) breathlessness score of 0.5 (0.0–2.0), and in the control group, the median (IQR) was 2.0 (1.0–3.0), (*p* = 0.042) (Table 2).

The cough score pre bronchoscopy comparing between the interventional and control groups are as follows, the median (IQR) in the interventional group was 1.0 (0.0–2.0); in the control group the median (IQR) was 1.0 (0.0–3.0); (*p* = 0.907). Post bronchoscopy median (IQR) score was 2.0(1.0–3.0) in the interventional group; and in the control group the median (IQR) score was 4.0 (2.3–5.0); (*p* = 0.001) (Table 2).

In analyzing the anxiety score, using the STAI, the interventional group scored a median (IQR) of 29.5 (26.3–40.8); while in the control group, median (IQR) 36.5 (33.5–43.5); (*p* < 0.001) (Table 2).

**Fig. 4:**
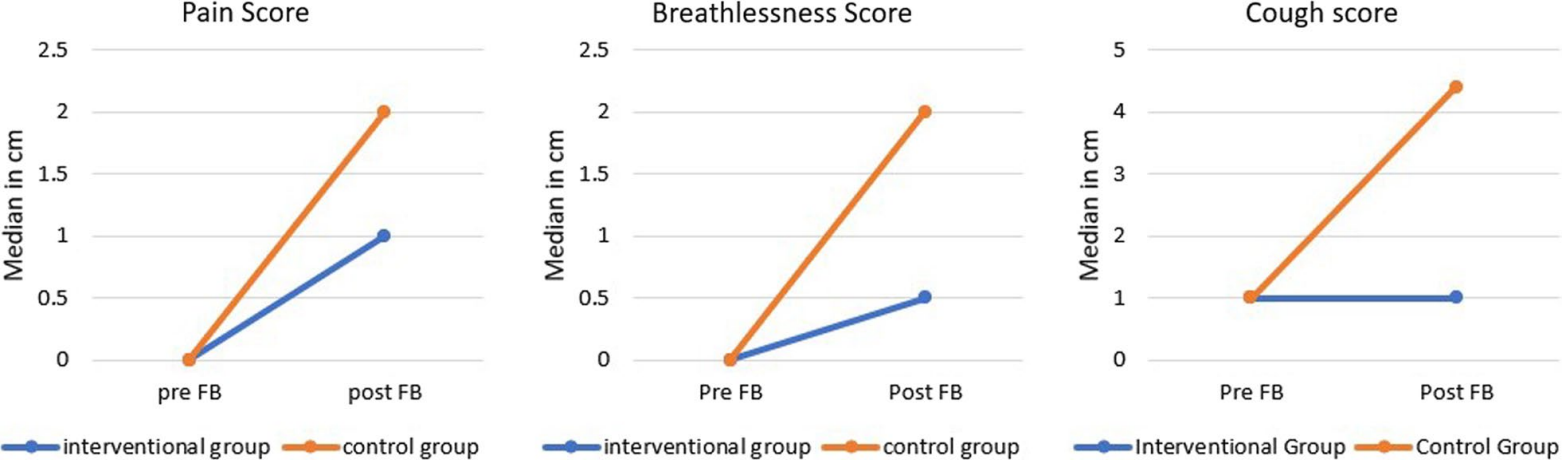
Comparison of median pain, breathlessness, and cough score between the interventional and control group, using a 10 cm VAS questionnaire

The figure below is a graphical representation of median pain, breathlessness, and cough score pre and post FB, measured using a VAS anchored at 10 cm. The control group showed a greater increase in median pain, breathlessness and cough scores compared to the interventional group post FB (Fig. 4).

With regards to the duration of FB, those in the interventional arm had shorter duration however this was not statistically significant. The majority of indication for FB was for bronchioalveolar lavage, this was true for both groups. The median sedation used in the intervention group compared to the control group were statistically similar (Table 3).

**Table 3:** Comparison of FB parameters between interventional and control group (*n* = 80)

Variables	n (%)	Group		*p*-value
		Intervention (n = 40)	Control (n = 40)	
**Indication of Bronchoscopy**				
BAL^µ^	70 (87.5)	33(82.5)	37 (92.5)	0.176^a^
BAL with TBLB/ EBB^¶^	10 (12.5)	7 (17.5)	3 (7.5)	
TBLB/EBB	0 (0)	0 (0)	0 (0)	
Duration of bronchoscopy* (minutes), median (IQR)		16.0 (12.8–24.5)	18.0 (15.0–20.0)	0.439^c^

**Sedation, median (IQR)**				
Midazolam (mg)	80	1.0 (1.0–2.0)	1.0 (1.0–2.0)	0.217^c^
Fentanyl (mcg)	80	50.0 (25.0–50.0)	37.5 (25.0–50.0)	0.257^c^

Statistical analysis was run using

^a^Chi-Square test of association

^c^Mann Whitney-U test

^µ^Broncho-alveolar lavage

^¶^Trans-bronchoscopy Lung biopsy/ Endo-bronchial Biopsy

## Discussion

This study evaluated the effectiveness of VR as an adjunctive treatment in FB, which serves as a distraction to the participant, to increase tolerability of FB. Our results showed that participants using VR during FB had lower sensations of breathlessness and cough compared to the control group. This suggests increased tolerability to FB in the VR group. Even though participants in the interventional arm did not show a significantly lower pain sensation than the control group, our analysis showed a lower median pain score reported by the intervention group.

Several studies have investigated the effect of distraction on patient tolerance of FB. Dette et al. published that distraction therapy with sights and sounds of nature significantly reduced pain during FB [6]. Dubois et al. reported that patients undergoing FB while listening to music were more comfortable and complained of less cough compared to the control group. Patients given appropriate audio-visual input as a method of distraction reported less anxiety and pain; as a result, took deeper breaths and complained of less dyspnea than control patients [20]. Jeppesen et al. studied the effects of listening to music before bronchoscopy in reducing anxiety, and concluded that listening to music reduces anxiety in patients undergoing bronchoscopy [21]. Playing music during FB improved physiological indicators of anxiety and decreased patient-perceived anxiety and pain [22]. Nilsson et al. reviewed 42 randomized clinical trials on music’s anxiolytic and analgesic effects during various clinical procedures and found positive benefits in about half of the trials [23]. Various studies conducted in the United Stated (US), China, and Europe reported positive effects of visual and audio distraction during other invasive procedures, including colonoscopy, sigmoidoscopy, interventional radiologic procedures, and burn dressing changes [24–30]. Dehghan et al. concluded that VR reduced anxiety in children in the perioperative setting [31].

Distraction is a method that uses pleasant stimuli to draw the patient's attention away from a painful and stressful situation. These stimuli include attractive and restful images, music, and soothing sounds [6]. Although the exact mechanisms of distraction is not fully understood, several hypotheses have been proposed. Music may stimulate the secretion of oxytocin, which results in psychological and physical relaxation [32]. Chan et al. found that music promoted relaxation in patients by reducing blood levels of adrenaline [33]. Music stimulates alpha brain waves leading to endorphins release, reducing anxiety, and promote relaxation [34]. Music helped regulate heart rate and blood pressure which are physiological measures of anxiety in patient undergoing colonoscopy [35]. Music therapy also decreased discomfort and increased the pain threshold and tolerance of medical procedures, acute and chronic pain [36]. Navidian et al. reported the effectiveness of distraction in increasing the tolerance threshold to FB (by reducing some of the signs such as pain, coughs, and dyspnea), shortening the length of the procedure, and enhancing patient satisfaction [16]. Using the Virtual reality device provided participants with an immersive experience which provided a more comprehensive method of distraction as patients were transported into their own virtual surrounding rendering them impervious to procedures and preparation leading up to flexible bronchoscopy.

The median time of FB was shorter by 2 min in the interventional group, however, this was not significant. Improved comfort, reduced cough, and breathlessness may have resulted in better patient tolerance and cooperation in the group receiving VR, enabling the operators to focus on the procedure and to complete it faster and efficiently. Midazolam and fentanyl were sedation used in participants undergoing flexible bronchoscopy, there were no significant difference between sedation used in the control group as compared to the interventional group. We postulate that sedation used did not play a significant role in reducing pain, breathlessness and cough in the interventional group comparing with the control group.

In addition, VR increased patient satisfaction with FB. The results of our study support the findings of other studies on the effect of distraction on patient satisfaction using various other methods. Klaming et al. reported that audio-visual distraction increased satisfaction by reducing cough, pain, and dyspnea associated with FB and improved patient tolerance toward the procedure. This also increased the likelihood of their return for a repeat procedure if required [26]. Angela Li et al. reported that VR has consistently been demonstrated to decrease pain, anxiety, unpleasantness, time spent thinking about pain, and perceived time spent in a medical procedure. In addition, healthcare providers have routinely commented that VR increases procedural cooperation while decreasing anxiety and distress [37]. In a recent study published by Lachker et al, distraction in the form of virtual reality hypnosis was found to reduce patients anxiety during bronchoscopy under local anesthesia, with a high level of satisfaction from patients, physicians and nurses. They postulated that hypnosis allows patients to be focused on their inner world, by including cognitive and behavioral components that help to influence body sensations and perception, and thus reducing patient anxiety undergoing invasive procedures [15]. We believe that hypnosis can be use as an adjunctive treatment concomitantly with virtual reality as a method of reducing pain and anxiety of participants undergoing invasive procedure.

Our study has some limitations. This study is a single-center study; as such, our participants were limited to a single bronchoscopist. A further multicenter study involving more bronchoscopists may be required to assess the effect of VR as an adjunct treatment. Another limitation is the possibility of recall bias of participants regarding answering post FB questionnaires; however, to minimize bias, we ensured that these questionnaires were answered only when the participants were fully conscious and orientated.

In conclusion, using VR in FB led to better satisfaction and tolerability compared to FB without VR. We also found significantly lower anxiety scores in the VR group. Our data suggest that VR as an adjunctive treatment in FB is effective and may be considered in all patients, especially those with high anxiety levels. We recommend a multicenter study to evaluate the use of VR in FB and its effectiveness in other invasive medical procedures.

## Data Availability

The data set used and/or analyzed during current study available from the corresponding author on reasonable request.

## References

[1] Günay E, Baki ED, Kokulu S, Ulaşlı SS, Öz G, Akar O (2015). Impact of multimedia information on bronchoscopy procedure: is it really helpful?. Ann Thorac Med.

[2] Miller RJ, Casal RF, Lazarus DR, Ost DE, Eapen GA (2018). Flexible bronchoscopy. Clin Chest Med.

[3] Du Rand IA, Blaikley J, Booton R, Chaudhuri N, Gupta V, Khalid S (2013). British Thoracic Society guideline for diagnostic flexible bronchoscopy in adults: accredited by NICE. Thorax.

[4] Hirose T, Okuda K, Ishida H, Sugiyama T, Kusumoto S, Nakashima M (2008). Patient satisfaction with sedation for flexible bronchoscopy. Respirology (Carlton, Vic).

[5] Ni YL, Lo YL, Lin TY, Fang YF, Kuo HP (2010). Conscious sedation reduces patient discomfort and improves satisfaction in flexible bronchoscopy. Chang Gung Med J.

[6] Diette GB, Lechtzin N, Haponik E, Devrotes A, Rubin HR (2003). Distraction therapy with nature sights and sounds reduces pain during flexible bronchoscopy: a complementary approach to routine analgesia. Chest.

[7] Lechtzin N, Rubin HR, Jenckes M, White P, Zhou LM, Thompson DA (2000). Predictors of pain control in patients undergoing flexible bronchoscopy. Am J Respir Crit Care Med.

[8] Prakash UB, Offord KP, Stubbs SE (1991). Bronchoscopy in North America: the ACCP survey. Chest.

[9] Fulkerson WJ (1984). Fiberoptic bronchoscopy. N Engl J Med.

[10] Lang EV, Benotsch EG, Fick LJ, Lutgendorf S, Berbaum ML, Berbaum KS (2000). Adjunctive non-pharmacological analgesia for invasive medical procedures: a randomised trial. Lancet (London, England).

[11] Tusek DL, Church JM, Strong SA, Grass JA, Fazio VW (1997). Guided imagery: a significant advance in the care of patients undergoing elective colorectal surgery. Dis Colon Rectum.

[12] Fernandez E (1986). A classification system of cognitive coping strategies for pain. Pain.

[13] Miller AC, Hickman LC, Lemasters GK (1992). A distraction technique for control of burn pain. J Burn Care Rehabil.

[14] Rusy LM, Weisman SJ (2000). Complementary therapies for acute pediatric pain management. Pediatr Clin North Am.

[15] Lachkar S, Gervereau D, Lanquetuit M, Thiberville L, Pradier HM, Roger M (2022). Hypnosis associated with 3D immersive virtual reality technology during bronchoscopy under local anesthesia. J Thorac Dis.

[16] Navidian A, Moulaei N, Ebrahimi Tabas E, Solaymani S (2018). The effect of audiovisual distraction on the tolerability of flexible bronchoscopy: a randomized trial. Clin Respir J.

[17] Spielberger C, Gorsuch R, Lushene R, Vagg PR, Jacobs G. Manual for the State-Trait Anxiety Inventory (Form Y1–Y2)1983.

[18] Nigussie S, Belachew T, Wolancho W (2014). Predictors of preoperative anxiety among surgical patients in Jimma University Specialized Teaching Hospital, South Western Ethiopia. BMC Surg.

[19] Dalal KS, Chellam S, Toal P (2015). Anaesthesia information booklet: Is it better than a pre-operative visit?. Indian J Anaesth.

[20] Dubois JM, Bartter T, Pratter MR (1995). Music improves patient comfort level during outpatient bronchoscopy. Chest.

[21] Jeppesen E, Pedersen CM, Larsen KR, Walsted ES, Rehl A, Ehrenreich J (2019). Listening to music prior to bronchoscopy reduces anxiety—a randomised controlled trial. Eur Clin Respir J.

[22] Triller N, Erzen D, Duh S, PetrinecPrimozic M, Kosnik M (2006). Music during bronchoscopic examination: the physiological effects. A randomized trial. Respir Int Rev Thorac Dis.

[23] Nilsson U (2008). The anxiety- and pain-reducing effects of music interventions: a systematic review. AORN J.

[24] Umezawa S, Higurashi T, Uchiyama S, Sakai E, Ohkubo H, Endo H (2015). Visual distraction alone for the improvement of colonoscopy-related pain and satisfaction. World J Gastroenterol.

[25] Lembo T, Fitzgerald L, Matin K, Woo K, Mayer EA, Naliboff BD (1998). Audio and visual stimulation reduces patient discomfort during screening flexible sigmoidoscopy. Am J Gastroenterol.

[26] Klaming L, Van Der Zwaag M, Minde D, Geraerdts H (2013). The influence of an audiovisual intervention on patient experience in a digital X-ray room. J Particip Med.

[27] Lee DW, Chan AC, Wong SK, Fung TM, Li AC, Chan SK (2004). Can visual distraction decrease the dose of patient-controlled sedation required during colonoscopy? A prospective randomized controlled trial. Endoscopy.

[28] Xiaolian J, Xiaolin L, Lan ZH (2015). Effects of visual and audiovisual distraction on pain and anxiety among patients undergoing colonoscopy. Gastroenterol Nurs Off J Soc Gastroenterol Nurses Assoc.

[29] Palakanis KC, DeNobile JW, Sweeney WB, Blankenship CL (1994). Effect of music therapy on state anxiety in patients undergoing flexible sigmoidoscopy. Dis Colon Rectum.

[30] Tan X, Yowler CJ, Super DM, Fratianne RB (2010). The efficacy of music therapy protocols for decreasing pain, anxiety, and muscle tension levels during burn dressing changes: a prospective randomized crossover trial. J Burn Care Res Off Publ Am Burn Assoc.

[31] Brown K, Foronda C (2020). Use of virtual reality to reduce anxiety and pain of adults undergoing outpatient procedures. Informatics.

[32] Nilsson U (2009). Soothing music can increase oxytocin levels during bed rest after open-heart surgery: a randomised control trial. J Clin Nurs.

[33] Chan MF (2007). Effects of music on patients undergoing a C-clamp procedure after percutaneous coronary interventions: a randomized controlled trial. Heart Lung J Crit Care.

[34] Almerud S, Petersson K (2003). Music therapy—a complementary treatment for mechanically ventilated intensive care patients. Intensive Crit Care Nurs.

[35] Smolen D, Topp R, Singer L (2002). The effect of self-selected music during colonoscopy on anxiety, heart rate, and blood pressure. Appl Nurs Res ANR.

[36] Kemper KJ, Danhauer SC (2005). Music as therapy. South Med J.

[37] Li A, Montaño Z, Chen VJ, Gold JI (2011). Virtual reality and pain management: current trends and future directions. Pain Manag.

